# Indicators of Subarachnoid Hemorrhage as a Cause of Sudden Cardiac Arrest

**DOI:** 10.5811/cpcem.2017.1.33061

**Published:** 2016-03-16

**Authors:** Joseph Zachariah, Jessica A. Stanich, Sherri A. Braksick, Eelco FM. Wijdicks, Ronna L. Campbell, Malcolm R. Bell, Roger White

**Affiliations:** * Mayo Clinic, Department of Neurology, Rochester, Minnesota; † Mayo Clinic, Department of Emergency Medicine, Rochester, Minnesota; ‡ Mayo Clinic, Department of Internal Medicine, Division of Cardiovascular Diseases, Rochester, Minnesota; § Mayo Clinic, Departments of Anesthesiology and Internal Medicine, Division of Cardiovascular Diseases, Rochester, Minnesota

## Abstract

Subarachnoid hemorrhage (SAH) may present with cardiac arrest (SAH-CA). We report a case of SAH-CA to assist providers in distinguishing SAH as an etiology of cardiac arrest despite electrocardiogram findings that may be suggestive of a cardiac etiology. SAH-CA is associated with high rates of return of spontaneous circulation, but overall poor outcome. An initially non-shockable cardiac rhythm and the absence of brain stem reflexes are important clues in indentifying SAH-CA.

## INTRODUCTION

Subarachnoid hemorrhage (SAH) as a cause of out-of-hospital cardiac arrest (OHCA) is rare.^1^–^3^ Most patients will present with pulseless electrical activity or asystole. SAH-CA is not typically due to intrinsic cardiac disease although numerous changes can be seen on electrocardiography.^4^,^5^ We report a case of SAH-CA to review the pathophysiology and remind clinicans to consider intracranial hemorrhage as a cause of death in any patient presenting with a non-shockable rhythm and lack of brainstem reflexes.

## CASE REPORT

A 55-year-old man with no cardiac history collapsed at work. Bystander cardiopulmonary resuscitation (CPR) was initiated promptly, and initial rhythm on emergency medical services (EMS) personnel arrival was asystole. Return of spontaneous circulation (ROSC) was achieved within four minutes of CPR and epinephrine administration. Endotracheal intubation was performed without sedative or paralytic agents and the patient was transported to the emergency department (ED). A prehospitalization 12-lead electrocardiogram (ECG) demonstrated diffuse anterolateral ST segment depression and aVR ST segment elevation (Image 1a). Upon arrival to the ED, the patient was found to be pulseless with electrical activity and required an additional seven minutes of CPR and multiple doses of epinephrine before ROSC was achieved. Point-of-care ultrasound demonstrated depressed left ventricular function without signs of right ventricular strain, aortic enlargement, or apical ballooning syndrome. The patient had fixed and dilated pupils, no spontaneous respiratory efforts, no response to painful stimuli, and required continuous epinephrine to maintain his blood pressure.

Based on the prehospital ECG, a posterior ST segment elevation myocardial infarction was considered as a cause of his arrest, although a repeat ECG demonstrated substantial resolution of the prehospital electrocardiographic abnormalities (Image 1b).

The case was discussed with the critical care cardiologist, and the cardiac catheterization laboratory was activated. The absence of an initial shockable rhythm raised diagnostic uncertainty and a computed tomography (CT) of the head was obtained prior to catheterization. CT of the head demonstrated diffuse SAH with intraventricular extension and mild hydrocephalus. Follow-up CT angiogram revealed a small anterior communicating artery aneurysm. The plan for coronary angiography was cancelled. Soon after admission to the neurosciences intensive care unit the patient was declared brain dead and became an organ donor.

## DISCUSSION

It is estimated that only 2–8% 1–3 of OHCA are caused by SAH. More than half of SAH-CA patients collapse without preceding headache and 12% may never reach medical attention.^6^–^9^ Non-cardiac causes of OHCA more often present with a non-shockable rhythm, like pulseless electrical activity (PEA) and asystole. Almost all patients with SAH have been shown to have ECG changes, and nearly two-thirds had changes suggestive of myocardial infarction or ischemia.^10^–^14^ ECG changes in the setting of SAH can include sinus tachycardia, atrial fibrillation, ST segment elevation or depression, QT prolongation, and rarely torsades de pointe, ventricular tachycardia and ventricular fibrillation (VF).^10^,^11^ VF after an initially non-shockable rhythm is likely secondary to CPR and epinephrine administration,^14^ VF and torsades de pointe in patients with OHCA secondary to SAH are uncommon.^4^,^10^,^11^,^14^

Anoxic ischemic brain injury from primary cardiac causes of sudden cardiac arrest preferentially affect the cortex and diencephalon and spare the brainstem.^15^–^17^ Absent pupillary, corneal, oculocephalic responses, spontaneous breathing or a Full Outline of UnResponsiveness (FOUR) score of 0 is highly unusual after CPR and ROSC unless associated with extremely prolonged resuscitation or exsanguination.^18^,^19^ A FOUR score is a 20-point clinical grading scale (with scores of 0 to 16) designed to assess patients with an impaired level of consciousness.^19^ The FOUR score assesses eye responses, motor responses, brainstem reflexes, and breathing pattern. It can be used in place of the Glasgow Coma Scale in intubated patients with traumatic or nontraumatic brain injuries and has been validated in the ED.^20^ The FOUR score would test several brainstem reflexes and breathing drive not identified with a GCS of 3.

**Image 2:**
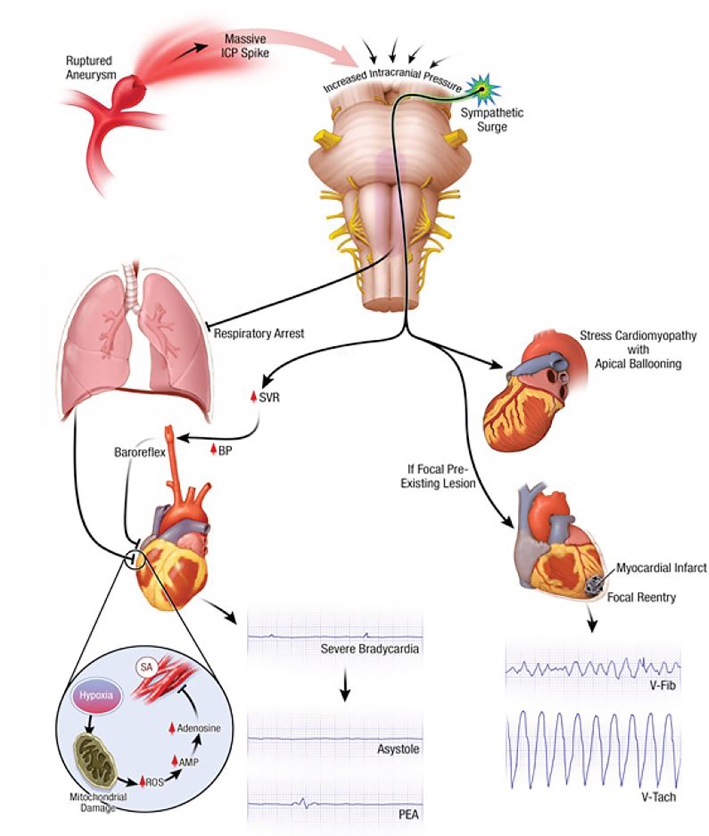
Pathophysiologic mechanism of cardiac arrest secondary to subarachnoid hemorrhage. Massive catecholamine release and sympathetic surge may stun cardiac tissue and/or cause an intense Cushing reflex; hypertension and bradycardia. A sudden massive increase in intracranial pressure may cause brainstem dysfunction and respiratory arrest from hypoxia. Severe hypoxia may trigger a cascade of biochemical changes, ultimately triggering endogenous release of adenosine, which causes profound bradycardia. *ICP,* Intracranial pressure; *ROS,* reactive oxygen species; *AMP,* activated protein kinase; *PEA,* pulseless electrical activity; *BP,* blood pressure; *SA,* striated muscle

Why does massive SAH lead to cardiac arrest? Why do patients with SAH-CA present with asystole or PEA? The cause of SAH-CA is not due to intrinsic cardiac disease, as coronary angiography and autopsy data have frequently shown patients have normal coronary arteries.^10^,^11^ In contrast, focal ischemia causes regional electrophysiological abnormalities and triggered automaticity, resulting in VF.^17^ The proposed pathophysiologic mechanism for cardiac arrest secondary to SAH is summarized in Image 2. Mechanisms for SAH-CA include (1) massive catecholamine release and sympathetic surge leading to cardiac stunning, or (2) sudden massive intracranial pressure (ICP) increase leading to brainstem dysfunction with respiratory arrest and hypoxia.^20^,^21^ The latter theory is supported in that the frequency of stress cardiomyopathy has been shown to be comparable among SAH patients regardless of cardiac arrest.^3^

A massive ICP surge results in a loss of brainstem reflexes leading to respiratory arrest and anoxia. Severe hypoxia triggers a cascade of biochemical changes, eventually triggering an endogenous release of adenosine acting on the heart to decrease contractility, atrioventricular conduction and pacemaker automaticity.^12^ Profound bradycardia and cardiac arrest can also occur as a result of an intense Cushing reflex.

A non-shockable rhythm in the setting of sudden cardiac arrest should raise suspicion for a primary non-cardiac etiology despite ECG changes suggestive of myocardial infarction or ischemia. The absence of brainstem reflexes should heighten suspicion for an intracranial process and an emergent head CT should be performed, given that a substantial proportion of cardiac arrest patients with non-shockable rhythms who achieve ROSC are patients with SAH.^3^,^5^ We propose that “hemorrhage” be added to the “H’s and T’s” differential for PEA or asystolic arrests originally described in the resuscitation guidelines.^17^ Remember that cardiorespiratory support should be maintained for brain death determination and possible organ donation, as SAH-CA is associated with poor outcomes. 1,21–23

## Figures and Tables

**Image 1a:**
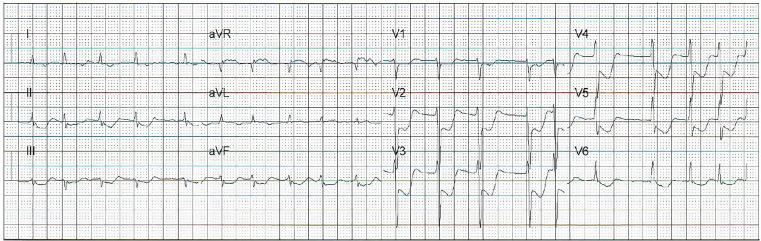
Post-resuscitation electrocardiogram on scene demonstrating atrial fibrillation with rapid ventricular rate of 114 bpm and diffuse ST depressions and ST elevation in aVR, in patient with cardiac arrest secondary to subarachnoid hemorrhage. *bpm,* beats per minute

**Image 1b:**
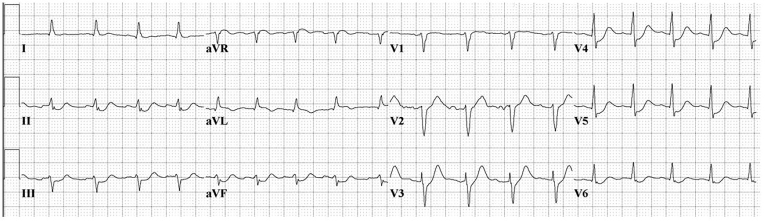
Electrocardiogram on admission showing normal sinus rhythm and substantial improvement in the prehospital electrocardiographic abnormalities.
